# Assessment of the Frequency of Online Searches for Symptoms Before Diagnosis: Analysis of Archival Data

**DOI:** 10.2196/15065

**Published:** 2020-03-06

**Authors:** Irit Hochberg, Raviv Allon, Elad Yom-Tov

**Affiliations:** 1 Institute of Endocrinology, Diabetes, and Metabolism Rambam Health Care Campus Haifa Israel; 2 Bruce Rappaport Faculty of Medicine Technion - Israel Institute of Technology Haifa Israel; 3 Microsoft Research Herzeliya Israel; 4 Faculty of Industrial Engineering and Management Technion - Israel Institute of Technology Haifa Israel

**Keywords:** search engines, diagnosis, screening

## Abstract

**Background:**

Surveys suggest that a large proportion of people use the internet to search for information on medical symptoms they experience and that around one-third of the people in the United States self-diagnose using online information. However, surveys are known to be biased, and the true rates at which people search for information on their medical symptoms before receiving a formal medical diagnosis are unknown.

**Objective:**

This study aimed to estimate the rate at which people search for information on their medical symptoms before receiving a formal medical diagnosis by a health professional.

**Methods:**

We collected queries made on a general-purpose internet search engine by people in the United States who self-identified their diagnosis from 1 of 20 medical conditions. We focused on conditions that have evident symptoms and are neither screened systematically nor a part of usual medical care. Thus, they are generally diagnosed after the investigation of specific symptoms. We evaluated how many of these people queried for symptoms associated with their medical condition before their formal diagnosis. In addition, we used a survey questionnaire to assess the familiarity of laypeople with the symptoms associated with these conditions.

**Results:**

On average, 15.49% (1792/12,367, SD 8.4%) of people queried about symptoms associated with their medical condition before receiving a medical diagnosis. A longer duration between the first query for a symptom and the corresponding diagnosis was correlated with an increased likelihood of people querying about those symptoms (rho=0.6; *P*=.005); similarly, unfamiliarity with the association between a condition and its symptom was correlated with an increased likelihood of people querying about those symptoms (rho=−0.47; *P*=.08). In addition, worrying symptoms were 14% more likely to be queried about.

**Conclusions:**

Our results indicate that there is large variability in the percentage of people who query the internet for their symptoms before a formal medical diagnosis is made. This finding has important implications for systems that attempt to screen for medical conditions.

## Introduction

Online self-diagnosis of health conditions is a well-known phenomenon that has grown substantially with ease of access to medical information facilitated by the internet and mobile technologies [1,2]. A large survey found that more than one-third of Americans self-diagnose when they encounter a health problem [3], and another study indicated that about 70% of American adults consult the internet for a variety of medical information [4].

The prevalence of self-diagnosis is leading countries and large epidemiologic centers to use the available information for public health goals [5,6]. Epidemics such as influenza and dengue fever have been tracked by observing the number of people who query internet search engines for the symptoms of these diseases [7,8]. A recent study showed the potential of identifying serious medical conditions, such as cervical and ovarian cancers, from people’s searches on online search engines [9]. These results suggest that search data could be used as a novel screening tool.

Nevertheless, utilizing search engines as an effective screening tool requires an accurate characterization of how people use search engines for self-diagnosis. In addition, conditions need to be independently characterized to understand the type and number of people who are searching for information and the common words used for these searches. It is currently not known how commonly people conduct an online search for their condition before diagnosis by a health professional. Determining this will provide an important indication of the percentage of people for whom online data screening is applicable and the diseases for which internet-based screening is effective. The purpose of this study was to characterize the prevalence and content of searches made by users before a medical diagnosis by a health care professional.

In this work, we analyzed data from search engine users who self-identified their diagnosis of a medical condition and traced back the data to determine how many of these instances could have been predicted by an earlier search for the signs and symptoms of the condition by that same user.

In addition, to better understand our search data results, we analyzed the association between the frequency of internet inquiries and the population’s general knowledge regarding certain conditions and their symptoms. We hypothesized that people inquire more about symptoms that they do not recognize or cannot associate with a certain disease.

## Methods

### Search Data

We extracted all queries made on Microsoft Bing in English by people in the United States between May 1, 2017, and April 30, 2018. For each user, we recorded an anonymized username, the time and date of the query, and the text used in the query. We focused on 20 medical conditions that are known to have evident symptoms, not systematically screened, not usually diagnosed in asymptomatic individuals by usual medical tests, and generally diagnosed after the investigation of specific symptoms. To ensure statistical power and validity, we limited the analysis to conditions for which at least 75 people self-identified their condition. The 20 conditions used in this study are listed in Table 1.

The population of self-identifying users was defined as those people who made a diagnosis ascertainment query (DAQ), indicating that they had been formally diagnosed with 1 of the 20 conditions analyzed in this study (eg, “I was diagnosed with COPD” or “I have COPD”). Queries that indicated the possibility of such a condition (eg, “do I have COPD”) were excluded. Specifically, DAQs were defined as queries that matched the phrases “I have” or “diagnosed with” and the name of the condition and excluded queries that contained any of the phrases “do I have,” “can I have,” “I think I have,” “did I have,” “nurse,” “patient,” “cat,” “dog,” “wife,” “husband,” “son,” or “daughter.”

For each condition, we calculated the fraction of people who queried about a relevant symptom before their first mention of the condition in the DAQ. The list of relevant symptoms was defined by two authors (IH and RA, both medical doctors) and enhanced using the synonym list developed by Yom-Tov and Gabrilovich [10].

In addition, symptoms were mapped to their perceived Medical Severity Rank (MSR), a validated measure of their apparent importance to both medical specialists and laypeople [11]. MSR measures the urgency of a symptom as perceived by people, from a symptom that requires immediate urgent care (MSR=1) to one that can be disregarded (MSR=10).

DAQs do not usually provide an indication of when the diagnosis was made. To estimate whether the DAQs are typically made around the time of diagnosis or throughout a person’s illness, we assessed if the time of the DAQ corresponded closely to the time of the first queries for hospitals, medical centers, or clinics. This analysis was conducted for each user indicating a diagnosis of 1 of the 3 malignant conditions (endometrial cancer, esophageal cancer, and lymphoma), where a hospital visit is usually required soon after the initial diagnosis is made.

**Table 1:** Conditions analyzed, symptoms associated with them, and the percentage of people who asked about the symptoms before their first query indicating that they have the condition.

Condition	Symptoms	People with symptoms^a^	
		n/N	%
Degenerative disc disease	Back pain, leg weakness, leg pain, leg numbness, leg tingling, loss of bowel control, and loss of bladder control	29/165	17.6
Chronic obstructive pulmonary disorder	Chronic cough, shortness of breath, dyspnea, recurrent pneumonia, wheezing, and dystonia	44/538	8.4
Menopause	Hot flash, night sweat, vaginal dryness, and alopecia	34/308	11.4
Heart failure	Shortness of breath, dyspnea, chronic cough, leg edema, leg swelling, rapid weight gain, and fatigue	116/896	12.9
Gout	Pain, tenderness, swelling, inflammation, and redness	520/2052	25.34
Ulcerative colitis	Diarrhea, abdominal pain, bloody bowel movement, rectal bleeding, tenesmus, lack of appetite, and fatigue	70/324	21.9
Bladder cancer	Blood in urine, hematuria, blood clots in urine, pain or burning sensation during urination, frequent urination, and not able to pass urine	33/302	11.3
Parkinson disease	Tremor and bradykinesia	144/3613	4.01
Endometrial cancer	Discharge and bleeding	25/110	22.7
Crohn disease	Diarrhea, blood in stool, fatigue, abdominal pain, cramping, mouth sores, reduced appetite, weight loss, and fistula	146/894	16.4
Angina pectoris or coronary heart disease	Chest pain, chest pressure, chest tightness, shortness of breath, and heartburn	239/775	30.8
Grave’s disease	Anxiety, irritability, heat sensitivity, increased sweating, weight loss, enlargement of thyroid or goiter, frequent bowel movements, diarrhea, bulging eyes, rapid heartbeat, rapid pulse, irregular heartbeat, and atrial fibrillation	112/479	23.4
Esophageal cancer	Difficulty or pain while swallowing solid food, vomiting, choking on food, heartburn, chest pressure, weight loss, coughing, and hoarseness	29/119	24.4
Lymphoma	Enlarged lymph nodes, night sweats, weight loss, intermittent fever, and fatigue	177/849	21.0
Plantar fasciitis	Foot pain and heel pain	10/212	5.2
Cellulitis	Painful area of skin, leg, foot, hand, or face; skin erythema or redness; skin edema; hot skin; and dropsy	4/278	1.8
Prostatitis	Painful, difficult, or frequent urination, blood in urine, groin pain, rectal pain, abdominal pain, low back pain, malaise, body aches, urethral discharge, and painful ejaculation or sexual dysfunction	12/103	12.6
Mastitis	Breast tenderness, pain or burning sensation, breast warmth or redness, breast swelling or thickening, breast lump, breast pain or burning, and malaise	10/90	12.2
Bell’s palsy	Facial paralysis on one side; drooping of the mouth to one side; asymmetrical mouth movement or smile; loss of blinking on one side; decreased or increased tearing; altered sense of taste; slurred speech; drooling; difficulty eating, drinking, or chewing; and pain or numbness behind the ear	6/168	3.6
Mononucleosis	Sore throat, malaise, headache, loss of appetite, myalgia, muscle pain, chills, and nausea	21/91	22.8

^a^Average=15.49% (1792/12,367).

### Survey Data

To estimate whether laypeople recognize the investigated medical conditions and their symptoms, we conducted a survey using the crowdsourcing platform CrowdFlower. We randomly selected 104 actual condition and symptom pairs and created another 156 random pairs. The latter were created by randomly matching a condition and a symptom and then verifying that the symptom is not manifested in the selected condition. A total of 10 crowdsourced workers were asked to answer, for each of the 260 pairs, whether they recognized the name of the medical condition and whether they thought that the given symptom could be the sign of the condition.

Laypeople’s knowledge about certain conditions and their symptoms, as determined through the surveys, was compared with the rate of searches for these symptoms in our search data.

### Statistics

Data analysis was conducted using MATLAB version 9.4.0. Spearman correlation was used to evaluate associations. The level of significance was set as 5% (*P*<.05). The Institutional Review Board of the Technion—Israel Institute of Technology approved this study.

## Results

On average, 618 people (mean SE 189) self-identified their diagnosis from 1 of the 20 analyzed conditions by making a DAQ. The users asked about each symptom, on average, 1.7 times. Table 1 lists the 20 conditions analyzed, the symptoms that were designated as being associated with that condition, and the percentage of people who queried about their symptoms before a formal diagnosis was made. On average, 15.49% (1792/12,367, SD 8.4%) of people queried about their symptoms before receiving a formal medical diagnosis.

There was a significant correlation between the percentage of people querying about a condition and the median number of days between the first symptom query and the DAQ (rho=0.60; *P*=.005; number of conditions=20). Thus, patients with conditions that had a longer duration between the onset of symptoms and the time of diagnosis were more likely to query for symptoms before they received a medical diagnosis.

We labeled each condition according to the lowest (most worrying) MSR for the related symptoms. We focused on the most worrying symptoms because of prior work, which shows that people most often recall the worst experience of pain [12,13], and thus, we hypothesized that people would be driven to search for the most worrying symptoms. A total of 4 conditions were excluded from this calculation because they had no symptoms for which Youngmann and Yom-Tov [11] provided an MSR. We found that more people inquired about the symptoms of a condition before a formal medical diagnosis (429/2328, 19.13%) for conditions that had an MSR≤2 as compared to those that had an MSR>2 (1382/10,107, 16.81%). Thus, people tended to query 14% more for diseases with more worrying symptoms (lower MSR) than those with less distressing symptoms.

A total of 4636 condition-symptom pairs were evaluated by crowdsourced workers in a survey aimed at estimating whether laypeople recognize the 20 investigated medical conditions and their symptoms. The responders reported recognizing the condition in 86% (3987/4636) of the pairs presented to them. The least recognized conditions were plantar fasciitis (40%, 16/40) and chronic obstructive pulmonary disorder (29%, 132/462). As described in the Methods section, our survey included actual disease-symptom pairs and sham disease-symptom pairs. For the real pairs, 86% (3987/4636) of responses correctly identified the pair as being associated. A similar level of success was observed for the sham pair, with 74% of the responses correctly indicating that the symptom was not associated with the disease.

Spearman correlation between the percentage of people querying for the symptom of a condition on Bing and the percentage of people who correctly recognized its symptoms in the survey was −0.47 (*P*=.08; number of conditions=14). This means that people tend to query more for conditions with less recognizable symptoms.

**Figure 1:**
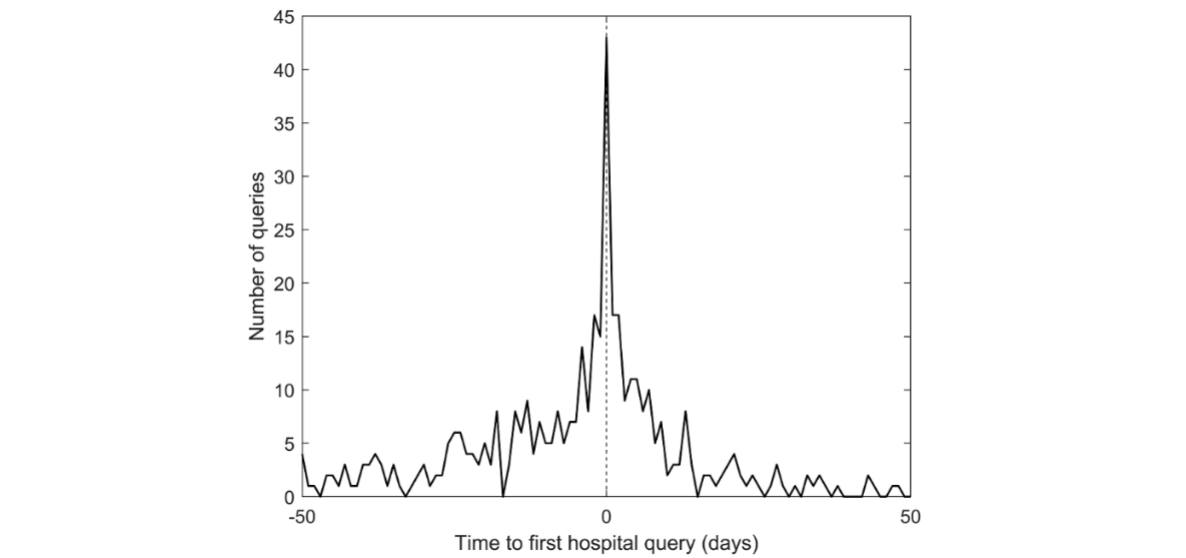
The time from the first query about a hospital and the time of the first query indicating the condition. Negative times indicate that the first query to a hospital was made prior to the first query about the disease. The figure shows data for endometrial cancer, esophageal cancer, and lymphoma.

To estimate whether the DAQs give an accurate indication of the time of initial diagnosis, we compared the timing of the DAQ with the first query about a hospital in the three malignant conditions. These specific conditions were analyzed because a hospital visit is usually required soon after the initial diagnosis is made. Table 2 shows the median time from the first query for a hospital or clinic until the first DAQ, the percentage of queries for hospitals made before the DAQ, and the percentage of people querying for a hospital. Figure 1 shows the distribution of the number of days between a query for a hospital and a query indicating a formal diagnosis, for all three conditions. There was no significant difference in time between queries for hospitals and queries for clinics (*P*>.05; rank sum test). As shown in Table 2 and Figure 1, there is only a short lag between the queries for a hospital and queries indicating a formal medical diagnosis. Indeed, 67% of queries for hospitals were within a window of 2 weeks before or after the DAQ (47% within a week around the DAQ). Figure 1 shows a clear peak for the first hospital searches occurring on the same day as the DAQ (19% of queries). Thus, queries indicative of diagnosis, specifically DAQs, correspond to the time of the actual diagnosis.

**Table 2:** The time from the first query for a hospital and the first query indicating the condition, the percentage of times the hospital query occurred before the diagnosis query, and the percentage of people who queried for a hospital.

Condition	Median time from hospital query to diagnosis query (days)^a^	Queries for hospitals made before queries for diagnosis^b^		People who inquired about a hospital^c^	
		n/N	%	n/N	%
Endometrial cancer	2.5	46/72	64	72/110	65
Esophageal cancer	14.3	45/66	68	66/119	55
Lymphoma	6.6	304/478	63.6	478/849	56.3

^a^Average=7.8.

^b^Average=65% (395/616).

^c^Average=59% (616/1078).

## Discussion

Internet interventions for public health have increased dramatically in the past decade, with multiple interventions across a wide range of conditions and populations. Following rapid developments in the last few years, it has been suggested that studies on internet interventions in the current decade will determine both the effectiveness and potential of such online interventions on public health [14].

It is well known that people search for symptoms on the internet before consulting a medical professional [15]. However, there has been no detailed characterization of when or what people inquire about when symptoms appear.

We found that an average of 15.5% of people queried for their symptoms before receiving a medical diagnosis from a health care professional, and we found high variability in this figure among the 20 conditions analyzed in our study. For instance, only 1.8% of patients diagnosed with cellulitis searched for a painful red area of skin before a medical diagnosis was made, compared with 30.8% of patients diagnosed with coronary heart disease who searched for chest pain or shortness of breath before receiving a medical diagnosis.

Our results showed that more people search for symptoms when the time between that first search to a formal diagnosis is longer (Spearman rho=0.6). One possible explanation for this correlation is that a long period of diagnosis gives an opportunity for more people to ask online about the condition. Another possible explanation is that people who search the internet for symptoms tend to do more thinking and consulting before approaching a medical professional, thus delaying their diagnosis. If the latter is true, better information needs to be provided to information seekers when it is likely that they have an acute condition that needs prompt treatment, emphasizing the need to enable internet-based screening for certain conditions.

The finding that most people inquire about hospitals around the time of making queries that suggest a diagnosis supports our assumption that such queries are made around the time of actual diagnosis. This concurs with past studies [16] that found that the number of people querying for different types of cancer were correlated with the incidence of cancers, not prevalence.

The survey results show that the conditions we studied and the match between the condition and symptom are well recognized. However, some conditions were less recognized than others. By comparing our survey results and our search data results, we showed a negative correlation between the rate of inquiry and the knowledge about a condition, suggesting that the less people know about a condition, the more they query for its symptoms. This result was not statistically significant (*P*=.08), but there is clearly a pattern that could illuminate one of the basic motives for people to first approach the Web when symptoms occur.

Our study limitations are inherent in a search engine data study, including the dependence on the user’s declaration of diagnosis, which assumes the exclusion of healthy people searching for diseases out of general curiosity. The main limitation is that most users do not declare the diagnosis in a search and therefore cannot be identified as patients. Moreover, users who self-declare their diagnosis are known to be a biased sample of patients, comprising relatively more females and younger people [17], a bias caused by a preference for query length. Thus, the prior rate of queries could be more heavily reflective of these population segments than that of the general population.

Although we have strived to find a comprehensive list of symptoms for each condition (including synonyms thereof), some symptoms could have been missed, especially colloquial references to symptoms, and these, if included, could have increased the reported percentage of searches for symptoms before the formal medical diagnosis. In addition, as our observation window is finite (1 year), people might have queried for their symptoms before the beginning of the data period. Such searches might increase the reported fraction of people performing searches for disease by approximately 1/12, meaning that the average percentage of people conducting an online search for a disease would be approximately 16.8%.

Finally, a symptom could be related to multiple underlying conditions. Although we have tried to focus on conditions with clear and distinct symptoms, such cases could have skewed our estimate for the search rates.

Despite these shortcomings, the strength of the study is significant; it is based on a diverse cohort made available because most people in industrialized countries now have access to the internet and use search engines as their primary data source when seeking health-related information [4]. This, together with our new understanding of when and how people seek information on symptoms, may enable future systems for screening of serious medical conditions from internet data, in general, and search queries, in particular, thereby overcoming the barriers of health illiteracy, unfamiliarity with medical conditions, and difficult access to the health system. Our hope is that such systems will enable earlier diagnosis of many serious medical conditions.

## Abbreviations

DAQ: diagnosis ascertainment query
MSR: Medical Severity Rank
